# Position of the Third Na^+^ Site in the Aspartate Transporter Glt_Ph_ and the Human Glutamate Transporter, EAAT1

**DOI:** 10.1371/journal.pone.0033058

**Published:** 2012-03-13

**Authors:** Turgut Bastug, Germano Heinzelmann, Serdar Kuyucak, Marietta Salim, Robert J. Vandenberg, Renae M. Ryan

**Affiliations:** 1 School of Physics, The University of Sydney, Sydney, Australia; 2 Faculty of Arts and Sciences, TOBB University of Economy and Technology, Ankara, Turkey; 3 Transporter Biology Group, Discipline of Pharmacology, School of Medical Sciences and Bosch Institute, The University of Sydney, Sydney, Australia; University of Cambridge, United Kingdom

## Abstract

Glutamate transport via the human excitatory amino acid transporters is coupled to the co-transport of three Na^+^ ions, one H^+^ and the counter-transport of one K^+^ ion. Transport by an archaeal homologue of the human glutamate transporters, Glt_Ph_, whose three dimensional structure is known is also coupled to three Na^+^ ions but only two Na^+^ ion binding sites have been observed in the crystal structure of Glt_Ph_. In order to fully utilize the Glt_Ph_ structure in functional studies of the human glutamate transporters, it is essential to understand the transport mechanism of Glt_Ph_ and accurately determine the number and location of Na^+^ ions coupled to transport. Several sites have been proposed for the binding of a third Na^+^ ion from electrostatic calculations and molecular dynamics simulations. In this study, we have performed detailed free energy simulations for Glt_Ph_ and reveal a new site for the third Na^+^ ion involving the side chains of Threonine 92, Serine 93, Asparagine 310, Aspartate 312, and the backbone of Tyrosine 89. We have also studied the transport properties of alanine mutants of the coordinating residues Threonine 92 and Serine 93 in Glt_Ph_, and the corresponding residues in a human glutamate transporter, EAAT1. The mutant transporters have reduced affinity for Na^+^ compared to their wild type counterparts. These results confirm that Threonine 92 and Serine 93 are involved in the coordination of the third Na^+^ ion in Glt_Ph_ and EAAT1.

## Introduction

Glutamate is the major excitatory neurotransmitter in the mammalian central nervous system. The extracellular concentration of glutamate is predicted to be as low as 25 nM [1] and is maintained by specific transport proteins called excitatory amino acid transporters (EAATs). Excessive extracellular glutamate is toxic for neurons, and therefore its concentration needs to be strictly controlled. Loss of this control due to dysfunction of EAATs has been implicated in several neurological diseases such as Alzheimer's disease, motor neuron disease and amyotrophic lateral sclerosis [2].

**Figure 1:**
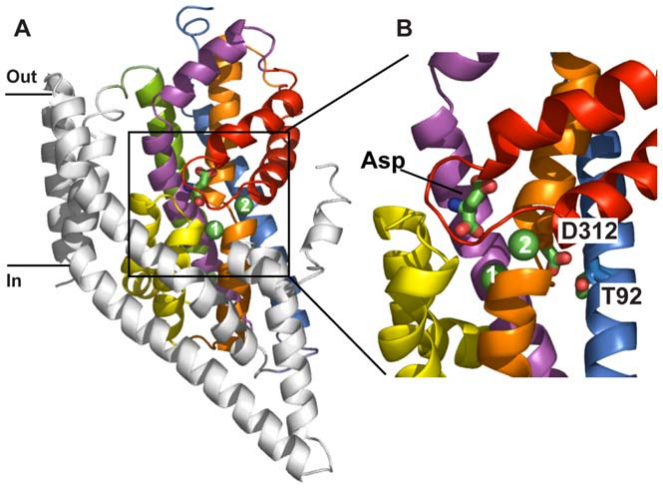
Structure of Glt_Ph_ with bound aspartate, Na1 and Na2 (PDB 2NWX). (A) One protomer in cartoon representation; transmembrane domain (TM)1, TM2, TM4 and TM5 (coloured in grey), TM3 (blue), TM6 (green), TM7 (orange), TM8 (magenta), hairpin (HP)1 (yellow) and HP2 (red). Bound aspartate is in stick representation and Na1(1) and Na2(2) are indicated as green spheres. (B) Close-up of the substrate binding site that has been rotated 90° around the vertical axis (coloured as in A). Aspartate 312 (D312) and threonine 92 (T92) are shown in stick representation. Figures were made using Pymol [39].

Glutamate transport via the EAATs is coupled to the co-transport of three Na^+^ ions and one H^+^ ion followed by the counter-transport of one K^+^ ion [3], [4]. Binding of Na^+^ and glutamate to the EAATs also activates an uncoupled Cl^−^ conductance [5]–[7]. A considerable amount of data has been gathered on the functional properties of the EAATs, but in the absence of any molecular structures it has been difficult to interpret this data and arrive at a molecular-level understanding of the transport mechanism of the EAATs. Determination of the crystal structure of a glutamate transporter homologue from *Pyrococcus horikoshii* (Glt_Ph_) [8] has therefore caused much excitement in the field. Glt_Ph_ is a Na^+^-dependent aspartate transporter that also has an uncoupled Cl^−^ conductance and the binding sites for the substrate aspartate and two Na^+^ ions have been identified [9], [10] (Figure 1A). Whether two or three Na^+^ ions are co-transported with each substrate molecule was not clear from the initial functional studies of Glt_Ph_, where a Hill coefficient >2 was observed [9], [11]. This issue has recently been resolved in an experiment using ^22^Na and ^3^H-aspartate, where it has clearly been shown that three Na^+^ ions are co-transported with each substrate as in the EAATs [12]. In this study, we follow the nomenclature already established and call the Na^+^ ion binding sites observed in the crystal structure Na1 and Na2 [9] and the third Na^+^ binding site Na3.

Glt_Ph_ shares about 36% amino acid sequence identity with the EAATs, which increases to above 60% for the residues forming the binding pocket [9]. Furthermore, many of the residues that have been implicated in substrate and ion binding, and also Cl^−^ permeation, in the EAATs are conserved in Glt_Ph_ [6], [13]–[17]. Thus, the Glt_Ph_ structure offers a good starting point for constructing homology models of the EAATs which can be used to perform simulations to gain a detailed understanding of the transport mechanism. All three Na^+^ ions need to be included in such studies, and this requires an accurate determination of the location of the Na3 site. Even a relatively small inaccuracy in the binding position of the ion could affect its coordination shell and lead to sizable inaccuracies in the calculated binding free energy. Several Na3 sites have been proposed from electrostatic calculations [18], [19] and molecular dynamics (MD) simulations [20], [21], which are compared with the available experimental data below.

The most important clue for the location of the Na3 site comes from the crystal structure of Glt_Ph_ [9], where the side chains of residues T92 and D312 are not involved in coordination of Na1 or Na2 (Figure 1B). This is in contrast to evidence from mutagenesis experiments on EAAT3 where both of these residues have been proposed to coordinate one of the coupled Na^+^ ions during glutamate transport [19], [22]. This suggests that the vicinity of the T92 and D312 side chain oxygens is the most likely place for the Na3 site and MD simulations of the Glt_Ph_ structure provide further clues in this regard. In MD simulations lasting 50 ns we have observed that, in the absence of a Na^+^ ion nearby, the D312 side chain swings ∼5 Å and coordinates Na1. Similar D312-Na1 coordination has been observed in other MD simulations of Glt_Ph_ [21]. The resulting coordination shell for Na1 is in substantial disagreement with that observed in the crystal structure, indicating that D312 should not be involved in the coordination of Na1. The only way the D312 side chain can be prevented from swinging towards Na1 in MD simulations is by allowing it to coordinate the third Na^+^ ion. Such a Na3 binding site was first proposed from electrostatic calculations, where four other oxygens besides the two oxygens from D312 were identified as possible ligands, namely, the side chains of Y88, T92 and N310, and the backbone of G404 [19]. This Na3 site was refined in subsequent MD simulations, where it was proposed to be formed by the side chains of T92, N310 and D312, and a water molecule [20].

The aim of this study was to carefully examine the proposed Na3 binding sites and find the best site that is consistent with all of the available data. Because the D312-Na3 coordination is fairly well established from both experiments [22] and simulations [20], we impose this condition which considerably simplifies the search. Our simulation results indicate that there is an alternate Na3 binding site near the one proposed in [20], which involves the side chains of T92, S93, N310, D312, and the backbone of Y89. This site provides a better coordination for the third Na^+^ ion and hence a lower binding free energy. We have used site-directed mutagenesis to change the proposed ligand residues T92, S93, N310 and D312 to alanine in Glt_Ph_. The N310A and D312A mutant transporters are non-functional, but functional analysis of T92A and S93A, and the equivalent residues in EAAT1, confirm their involvement in the coordination of Na3.

## Results

### MD simulations of Glt_Ph_ reveal a novel Na3 site

To date, all MD simulations of Glt_Ph_ and predictions for the Na3 site have used the aspartate-bound ‘closed’ structure. In this study, we employ two simulation systems derived from the aspartate-bound ‘closed’ (PDB: 2NWX) and the TBOA-bound ‘open’ (PDB: 2NWW) structures. In the closed structure the helical hairpin 2 (HP2) gate is closed and aspartate and two Na^+^ ions (Na1 and Na2) are bound (2NWX). While in the open structure the non-transportable inhibitor TBOA is bound in place of aspartate which prevents closing of the HP2 gate and only one Na^+^ ion (Na1) is bound (2NWW). We have created an open structure from 2NWX by removing the ligands and observing the opening of the HP2 gate in MD simulations [23], [24]. A similar apo structure has been created from 2NWW by removing TBOA. To distinguish between these two structures, we will refer to them as “closed” and “open” following their origin, although in both structures the HP2 gate is open.

**Figure 2:**
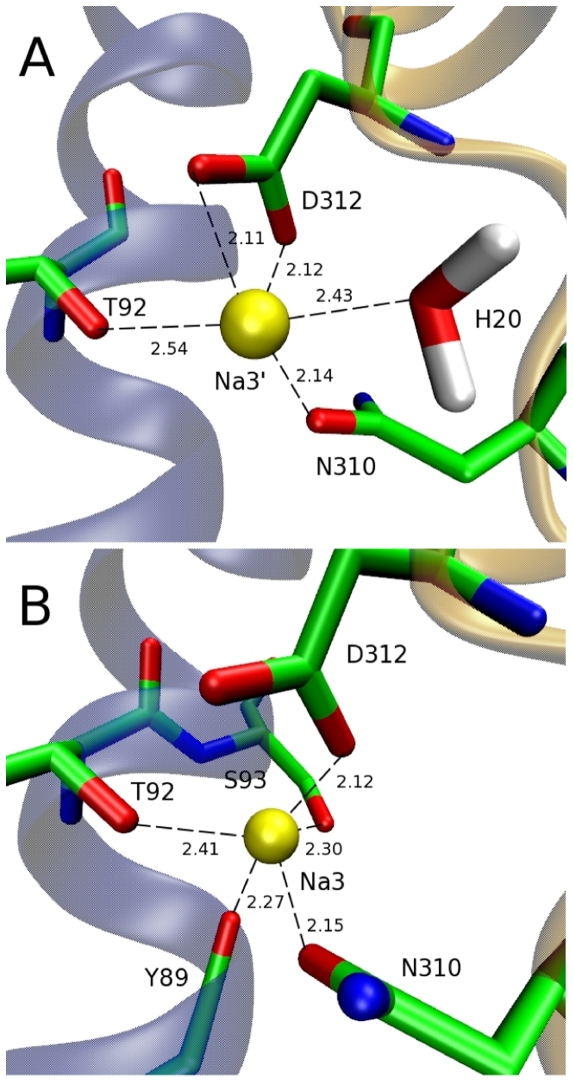
Comparison of the Na3 and Na3′ sites. Snapshots of the Na3′ (A) and Na3 (B) sites showing the oxygens (red) involved in the coordination of a Na^+^ ion (yellow). Note the flipping of the N310 side chain between (A) and (B). Distances between the Na^+^ ion and coordinating ligands are shown in angstroms.

As T92 and D312 have been proposed to bind a Na^+^ ion in EAAT3 [19], [22] and they are unlikely to interact with Na1 or Na2, we placed a Na^+^ ion in the vicinity of the T92 and D312 side chains in the system obtained from the closed structure, and equilibrated this system in MD simulations. The resulting coordination shell is consistent with that found previously [20] and consists of the oxygens from the sidechains of T92, N310 and D312 (two), and a water molecule (this site will be called Na3′ henceforth, see Figure 2A). We next considered the system obtained from the open structure of Glt_Ph_. Surprisingly, when this system is equilibrated in MD simulations with a Na^+^ ion placed near the T92 and D312 side chains, a rather different coordination shell emerges, consisting of the oxygens from the side chains of T92, S93, N310, D312 (one oxygen), and the backbone of Y89 (Figure 2B). In the new site (to be called Na3), water and one of the D312 carboxyl oxygens in the Na3′ site are replaced with two new ligands, which leads to a tighter Na^+^ coordination shell due to shorter Na-O distances.

**Figure 3:**
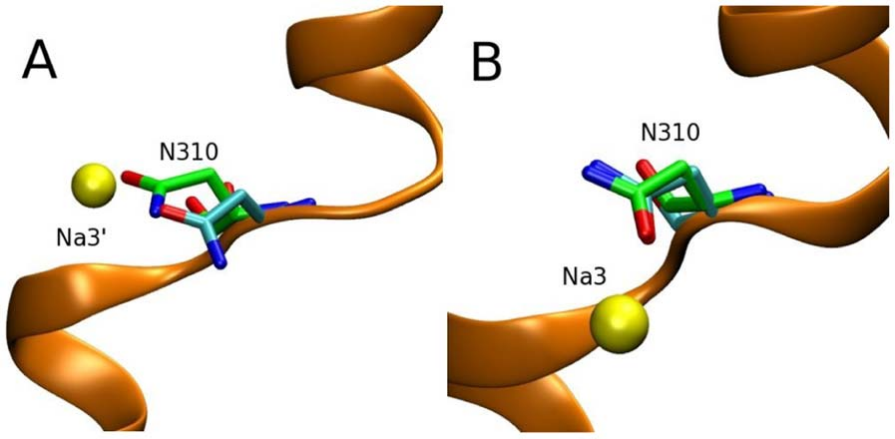
The N310 configurations in the closed and open structures contrasted with the MD results. Comparison of the N310 configuration from the crystal structure (green backbone) with that obtained from MD simulations (cyan backbone). Na^+^ ions are shown as yellow spheres. (A) Closed structure (PDB 2NWX) vs. Na3′ site, (B) Open structure (PDB 2NWW) vs. Na3 site.

An immediate question is why there are two different Na3 sites if the two structures are so similar. The answer becomes obvious when the open and closed crystal structures are superimposed—the O and N atoms in the N310 side chain are interchanged in the two structures (Figure 2A,B). This different position of the N310 side chain leads to a difference of about 2 Å between the position of Na3 and Na3′. Na3 is closer to transmembrane domain 3 (TM3), where the extra coordinating ligands reside. The aspartate and TBOA bound crystal structures were solved at ∼3 Å and ∼3.5 Å respectively. At this resolution it is difficult to assign the positions of side chains with accuracy so we have performed several tests to see which of the two positions of the N310 side chain is more realistic. To check the stability of the respective binding sites, we have equilibrated the open and closed structures without Na^+^ ions and Asp. In 5 ns MD simulations, the N310 side chain has retained its original configuration in both the open and closed structures, indicating that there is no intrinsic preference for one of the configurations in the ‘apo’ state. Next, we have equilibrated the two structures in the presence of a Na^+^ ion in the respective Na3 binding sites. Snapshots obtained from the MD simulations are compared to the respective crystal structures (Figure 3). Introduction of the Na^+^ ion at Na3′ is seen to cause a relatively large perturbation of the N310 side chain in the closed structure, e.g., the Cα atom is displaced by about 2 Å from its crystal structure position (Figure 3A). In contrast, placement of a Na^+^ ion at Na3 induces very little change in the N310 side chain configuration in the open structure (Figure 3B). If there is an unobserved third Na^+^ ion in these crystal structures, its introduction in the correct location in the MD simulations should cause minimal disruption to the coordinating ligands, which is the case for the Na3 site but not for Na3′.

To ensure that the N310 side chain conformation is the only difference between the 2NWX and 2NWW structures with regard to the Na3 binding site, we have repeated the MD simulations with these two structures, but with the O and N atoms in the N310 side chain flipped in each case. The average Na^+^–O distances listed in Table 1 essentially reveal an identical coordination shell structure in both cases, as long as the N310 side chains have the same conformation. Finally, we have calculated the free energy of binding of a Na^+^ ion to the Na3 and Na3′ sites in the absence of other ions and substrate. An example of the thermodynamic integration (TI) calculations illustrating the convergence of the binding free energy for the Na3 site is shown in Figure S1. The binding free energy is found to be 4 kcal/mol lower for the Na3 site compared to that of Na3′ (Table 2), which again supports Na3 as the correct binding site. To summarize the evidence presented, the Na3 site is more consistent with the crystal structure compared to the Na3′ site and leads to a lower binding free energy. Therefore, the Na3 site identified in this study is the most likely binding site for the third Na^+^ ion in Glt_Ph_.

**Table 1:** Coordination of the third Na^+^ ion showing the Na-O distances (in Å) for the closed (2NWX), and open (2NWW) structures, which give rise to the Na3′ and Na3 binding sites, respectively.

	Na3′		Na3	
	2NWX	2NWW*	2NWW	2NWX*
Y89 (O)	3.8±0.3	3.9±0.3	2.3±0.1	2.3±0.1
T92 (OH)	2.4±0.1	2.4±0.1	2.4±0.1	2.4±0.1
S93 (OH)	4.3±0.2	4.1±0.3	2.3±0.1	2.4±0.1
N310 (OD)	2.2±0.1	2.2±0.1	2.2±0.1	2.2±0.1
D312 (O1)	2.2±0.1	2.2±0.1	2.1±0.1	2.1±0.1
D312 (O2)	2.2±0.1	2.2±0.1	3.5±0.3	3.6±0.2
H2O (O)	2.4±0.2	2.4±0.2		

When the N310 side chain is flipped in the closed structure (2NWX*), the same coordination shell is observed as in the open structure (2NWW). Similarly, when the N310 side chain is flipped in the open structure (2NWW*), an identical Na coordination to that of 2NWX is observed.

**Table 2:** Binding free energies for Na^+^ ions in various locations (in kcal/mol).

Ligand	ΔG_int_	ΔG_tr_	ΔG_b_
Na3	−23.3±1.2	4.6	−18.7±1.2
Na3′	−19.3±0.9	4.6	−14.7±0.9
Na1	−16.2±1.3	4.9	−11.3±1.3
Na1 (Na3)	−11.9±1.3	4.8	−7.1±1.3

The interaction energy (ΔG_int_), entropic contributions (ΔG_tr_) and the total binding energy (ΔG_b_) are listed separately. The interaction free energy differences are obtained from the average of the forward and backward transitions. Presence of a second Na^+^ ion is indicated in parentheses. Errors are estimated from block data analysis using 50 ps windows. The Na2 site is not considered because it is formed only after the HP2 gate shuts.

### Order of binding of Na^+^ and aspartate to Glt_Ph_

The binding order of Na^+^ ions and aspartate provides more evidence in this regard. It has been argued that, because access to the Na3 site is through the Na1 site and the path is very narrow, the Na3 site must be occupied first otherwise a Na^+^ ion would not be able to access the Na3 site [20]. Here we provide evidence from the binding free energy calculations in favour of this proposal. As indicated in Table 2, the free energy of binding of a Na^+^ ion is 7 kcal/mol lower for the Na3 site compared to the Na1 site. The large free energy difference between these two sites suggests that once a Na^+^ ion binds to Na3 there is a higher probability it will remain bound than if it were to bind to Na3′ where the free energy difference compared to Na1 is smaller (3 kcal/mol). The higher free energy of binding of Na^+^ to Na3 suggests that this site is a more stable one compared to Na3′.

A related question is whether aspartate binds before or after Na1. As shown in Table 2, the second Na^+^ ion binds to the Na1 site with a fairly large free energy (−7.1 kcal/mol) in the absence of aspartate. When aspartate is included in the MD simulations following the binding of two Na^+^ ions at the Na3 and Na1 sites, the resulting system is extremely stable – all three aspartate molecules in the trimer have been observed to remain in their binding pockets for more than 50 ns. However, once the Na^+^ ion is removed from the Na1 site, aspartate becomes unstable and dissociates – all three aspartate molecules in the trimer have been observed to move away from their binding pockets within 5 ns of MD simulations. These observations provide strong evidence that aspartate binds only after the binding of two Na^+^ ions. Thus the proposed binding order of the ligands to the transporter is Na3, Na1, aspartate and Na2.

### The Na3 site in the archaeal aspartate transporter Glt_Ph_

**Figure 4:**
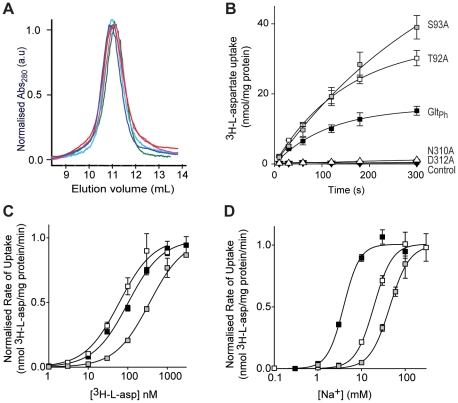
T92A and S93A mutations in Glt_Ph_ have reduced Na^+^ affinity. (A) Size exclusion column profile for wild type Glt_Ph_ (dark blue), T92A (red), S93A (green), N310A (pink) and D312A (cyan). (B) Uptake of 100 nM ^3^H-L-aspartate in the presence of 100 mM NaCl for Glt_Ph_ (black squares), T92A (white squares), S93A (grey squares), N310A (white triangles) and D312A (black triangles). Control levels are from uptake performed in the presence of internal buffer (100 mM KCl, 20 mM HEPES/Tris pH 7.5) (C) ^3^H-L-aspartate concentration-dependent transport in the presence of 100 mM NaCl by Glt_Ph_ (black squares), T92A (white squares) and S93A (grey squares). (D) Na^+^ concentration-dependent transport of 100 nM ^3^H-L-aspartate for Glt_Ph_ (black squares), T92A (white squares) and 500 nM ^3^H-L-aspartate for S93A (grey squares). Data in (C) and (D) are normalised to the maximal velocity of transport and all data are from the mean of at least 3 separate experiments ± s.e.m.

To confirm the alternate Na3 binding site described above, site-directed mutagenesis was performed on the Na3 coordinating residues T92, S93, N310 and D312. Y89 contributes a backbone carbonyl oxygen to the Na3 site and therefore was not investigated by mutagenesis. All mutations were well tolerated by Glt_Ph_; similar protein yields were obtained and all mutant transporters eluted as symmetrical peaks following size exclusion chromatography at the same point as wild type Glt_Ph_ (Figure 4A). Purified protein was reconstituted into liposomes and function was assayed by examining ^3^H-L-aspartate uptake.

The N310A and D312A transporters did not exhibit ^3^H-L-aspartate uptake over background levels and were not investigated further (Figure 4B). This is not surprising as mutation of either of these residues in EAAT2 results in a non-functional transporter [25]. In addition, these two residues are in the highly conserved NMDGT motif that contains residues important for substrate binding [9]. Both T92A and S93A are functional aspartate transporters (Figure 4B) but display altered aspartate affinity and changes in the Na^+^ dependence of transport. T92A transports aspartate with a slightly higher affinity than wild type Glt_Ph_; *K*_0.5_ of 61 ±10 nM compared to 100±10 nM for wild type Glt_Ph_. In contrast, S93A exhibits a reduction in aspartate affinity with *K*_0.5_ of 370±60 nM (Figure 4C). The Na^+^ dependence of aspartate transport was measured by monitoring the uptake of ^3^H-L-aspartate under a range of Na^+^ concentrations, from 0.1 mM to 300 mM. Both T92A and S93A showed a reduction in Na^+^ affinity compared to wild type Glt_Ph_; T92A *K*_0.5_ of 19±1 mM and S93A *K*_0.5_ of 44±2 mM compared to Glt_Ph_ *K*_0.5_ of 3.9±0.4 mM (Figure 4D) but there was no change in the Hill coefficient (Glt_Ph_, 2.3±0.5; T92A, 2.1±0.2; S93A, 1.9±0.2). As T92 and S93 are not close enough to coordinate Na1 or Na2, we propose that these mutations are affecting the Na3 site. Indeed, when the binding free energy of Na3 is calculated in the presence of the T92A and S93A mutations, it is reduced by 7.5 kcal/mol and 5.9 kcal/mol respectively, while the binding free energy of Na1 is unaltered (Table 3). Interestingly, the maximal velocity of ^3^H-L-aspartate transport measured in the presence of 100 mM Na^+^ and saturating substrate concentrations by T92A is 42±1 nmol/mg protein/min and S93A is 58±3 nmol/mg protein/min. These rates are increased compared to the maximal velocity of ^3^H-L-aspartate transport by Glt_Ph_ (7.5±0.5 nmol/mg protein/min) (Figure S2). The increased rate of transport observed for the mutant transporters could be due to the reduced affinity of Na3 for its binding site. Na3 may unbind more easily from T92A and S93A than wild type Glt_Ph_ and thus facilitate the turnover of the transporter. Taken together, the simulation and experimental results strongly support a role for T92 and S93 in coordinating Na3 in Glt_Ph_.

**Table 3:** Effect of the T92A and S93A mutations on the binding free energies of Na^+^ ions at the Na3 and Na1 sites (in kcal/mol).

Ligand	Glt_Ph_	T92A	S93A
Na3	−18.7±1.2	−11.2±1.4	−12.8±1.2
Na1 (Na3)	−7.1±1.3	−6.7±1.2	−6.4±1.4

Mutations significantly reduce the binding free energies of Na3 but not of Na1. Presence of a second Na^+^ ion is indicated in parentheses.

### The Na3 site in the human glutamate transporter EAAT1

**Figure 5:**
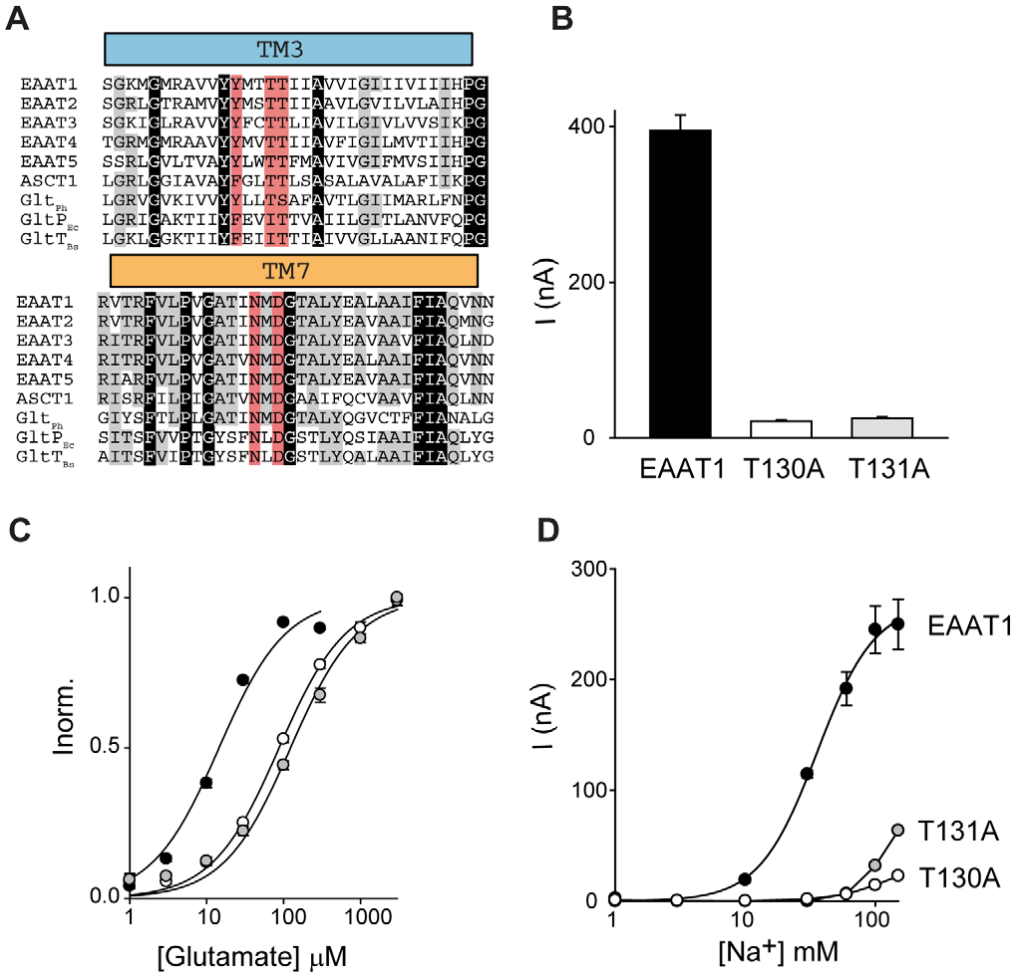
T130A and T131A mutations in EAAT1 have reduced Na^+^ affinity. (A) Amino acid alignment of TM3 and TM7. Alignment was made using ClustalW2 [40] and adjusted manually. Amino acid sequences are; human (h)EAAT1 (NP_004163.3), hEAAT2 (NP_004162.2), hEAAT3 (NP_004161.4); hEAAT4 (NP_005062.1); hEAAT5 (NP_006662.3); hASCT1 (NP_003029.2); *Pyrococcus horikoshii* Glt_Ph_ (NP_143181); *Escherichia coli* GltP_Ec_ (EGT70436.1); *Bacillus stearothermophilus* GltT_Bs_ (P24943.1). Red shading indicates the residues that form the Na3 site, grey shading indicates conserved residues and black shading indicates residues absolutely conserved. (B) Average current activated by the application of 100 µM L-glutamate to oocytes clamped at −60 mV expressing EAAT1 (black), T130A (white) and T131A (grey). (C) L-glutamate concentration-dependent currents for EAAT1 (black), T130A (white) and T131A (grey). (D) Na^+^ concentration-dependent currents in the presence of 300 µM L-glutamate for EAAT1 (black circles), and 1 mM L-glutamate for T130A (white circles) and T131A (grey circles). Data in (C) are normalised to the current at saturating glutamate concetrations and all data are from the mean of at least 3 separate experiments ± s.e.m.

Glt_Ph_ shares ∼36% amino acid identity with the EAATs and this conservation is even higher in the transport domain consisting of hairpin (HP)1, transmembrane domain (TM) 7, HP2 and TM8 where amino acid identity increases to ∼60%. The recent crystal structure of the inward facing Na^+^ and aspartate bound Glt_Ph_ revealed that TM3 and TM6 are also part of the transport domain core that is proposed to move over 18 Å during the transport cycle [26]. TM3 is not as highly conserved as the rest of the transport domain, Glt_Ph_ shares ∼40% amino acid identity with EAAT1 in this region, but there are several residues that are conserved throughout the Na^+^ dependent transporters of this family including Glt_Ph_, EAAT1-5 and the neutral amino acid transporter, ASCT1. T92 is highly conserved, while S93 is a threonine residue in the rest of the family (Figure 5A). Interestingly, T92 is replaced with an isoleucine residue in the H^+^ dependent glutamate transporters from *Bacillus stearothermophilus* (GltT_Bs_) and *Esherichia coli* (GltP_Ec_). To investigate if the third Na^+^ site predicted by simulation and experimental studies on Glt_Ph_ is conserved in the human glutamate transporters the T130A (equivalent to T92) and T131A (equivalent to S93) mutations were introduced into EAAT1. The residues equivalent to N310 and D312 were not investigated as these transporters have been shown to be non-functional in EAAT2 [25].

Application of 100 µM L-glutamate to oocytes expressing EAAT1 elicits a current of 395±20 nA when the oocytes are clamped at −60 mV (Figure 5B). Application of 100 µM L-glutamate to oocytes expressing the mutant transporters T130A and T131A also activates a conductance at −60 mV but it is reduced in amplitude to 21±2 nA and 25±2 nA for T130A and T131A respectively (Figure 5B). To further investigate the impact of the T130A and T131A mutations in EAAT1 the *K*_0.5_ for L-glutamate transport and the *K*_0.5_ for the Na^+^-dependence of L-glutamate transport were determined. Oocytes expressing EAAT1, T130A or T131A were clamped at −60 mV and increasing doses of L-glutamate were applied. For wild type EAAT1 the *K*_0.5_ value for L-glutamate is 15±1 µM (Figure 5C). In contrast, the L-glutamate affinity for T130A and T131A is reduced with *K*_0.5_ values of 87±3 µM and 124±11 µM, respectively (Figure 5C).

To investigate the Na^+^-dependence of transport the current elicited by saturating L-glutamate concetrations (300 µM for EAAT1 and 1 mM for T130A and T131A) in the presence of increasing amounts of Na^+^ was determined. For wild type EAAT1 the *K*_0.5_ for Na^+^ is 28±4 mM and the Hill coefficient is 2.8±0.4. The Na^+^ dependence of L-glutamate transport for T130A and T131A was significantly altered compared to wild type EAAT1 (Figure 5D). For both mutant transporters, the highest Na^+^ concentration tolerated by *Xenopus laevis* oocytes (150 mM) was not saturating and the data could not be fit to the Hill equation to determine a *K*_0.5_ for Na^+^ or a Hill coefficient. Nevertheless, it is evident that both of these mutations have significantly reduced the ability of Na^+^ to support glutamate transport.

## Discussion

Molecular dynamics is a powerful tool that can simulate movements and binding events from static snapshots of proteins obtained from X-ray crystallography, but functional studies are vital to confirm these predictions and maximise the information obtained from MD simulations. In this study, we have combined these techniques to explore the third Na^+^ binding site in the glutamate transporter family using Glt_Ph_ and EAAT1 as representative transporters.

The crystal structure of the aspartate transporter Glt_Ph_ with bound substrate and two Na^+^ ions was an important advance in our understanding of the transport mechanism of the glutamate transporter family [9]. Substrate transport by the human glutamate transporters EAAT2 [3] and EAAT3 [4], and also by Glt_Ph_ [12], is coupled to the co-transport of 3 Na^+^ ions, yet only 2 Na^+^ binding sites were identified in the Glt_Ph_ structure [9] leaving the location of the third Na^+^ ion unknown. Previous studies using electrostatic calculations, MD simulations and mutagenesis studies have proposed several different sites for Na3 [18]–[22]. Here, we present a new location of the Na3 site as determined by MD simulations of both the open and closed Glt_Ph_ structures. The veracity of this site has been further tested by calculating the binding free energy of substrate and ions and site-directed mutagenesis experiments, which corroborate our proposed Na3 location.

**Figure 6:**
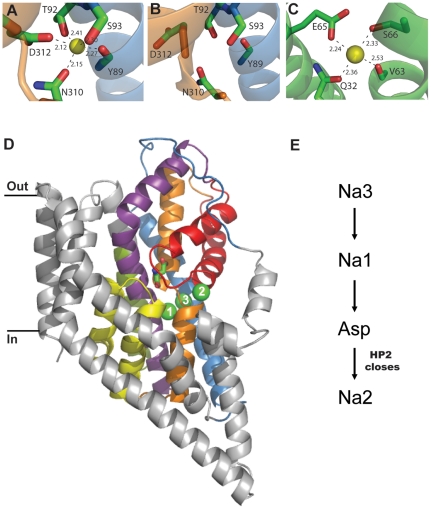
The third Na^+^ binding site in Glt_Ph_. (A) The coordination of the Na3 binding site identified in this study. TM3 (blue) and TM7 (orange) are shown in cartoon and the coordinating residues are shown in stick representation and labelled. Na3 is shown as a yellow sphere. (B) The area of the Na3 site in the inward occluded structure of Glt_Ph_ (PDB 3KBC); colouring as in A. (C) Na^+^ binding site on the rotor ring (c ring) of the F-Type Na^+^ ATPase from *Ilyobacter tartaricus* (PDB 1YCE). V63, S66 are from the A subunit and Q32, E65 are from the B subunit of the c ring. Na^+^ ion is shown as a yellow sphere. (D) One protomer of Glt_Ph_ with bound aspartate, Na1, Na2 and Na3. The transport domain is shown in colour; TM3 (blue), TM6 (green), HP1 (yellow), TM7 (orange), HP2 (red) and TM8 (purple) and the 3 Na^+^ ions are shown as green spheres and numbered. (E) The proposed order of binding for substrate and ions to Glt_Ph_. All distances are in angstroms and structure figures were made using Pymol [39].

The binding site for Na3 is made up of residues from TM3 and TM7 which are both in the transport domain that is proposed to undergo a large conformational change during substrate translocation [26]. T92 (TM3), S93 (TM3), N310 (TM7) and D312 (TM7) all donate side chain oxygens to the binding site while Y89 (TM3) contributes a backbone carbonyl oxygen (Figure 6A). To verify the contribution of the side chains of T92, S93, N310 and D312 to the Na3 site predicted by MD simulations we used site-directed mutagenesis to change these residues in Glt_Ph_ to alanine. The N310A and D312A transporters were non-functional. These residues reside in the highly conserved NMDGT motif that contains residues important for substrate binding [9] and mutations of the equivalent residues in EAAT2 leads to non-functional transporters [25]. In contrast, the T92A and S93A transporters were functional aspartate transporters but these mutations result in a reduced affinity for Na^+^ compared to wild type Glt_Ph_, while the Hill coefficient is unaltered. This data suggests that the ability of Na^+^ to support aspartate transport is diminished but the number of Na^+^ ions coupled to transport is unchanged. There is also some variation in the affinity of aspartate for these mutant transporters. T92A has a slightly higher aspartate affinity, while S93A displays a reduced aspartate affinity compared to wild type Glt_Ph_. As aspartate binding and transport is coupled to Na^+^, it is not surprising that disruption of the Na3 site also affects the *K*_0.5_ for aspartate transport. Further evidence to support the location of Na3 comes from the binding free energies calculated when the T92A and S93A mutations were introduced into Glt_Ph_. Both of these mutations reduced the binding free energy calculated for Na3, but did not affect the binding free energy calculated for Na^+^ binding to the Na1 site (Table 3). To translate the information gained about the location of the Na3 site in Glt_Ph_ to the human glutamate transporters, parallel mutagenesis experiments were performed in EAAT1. The residues corresponding to T92 and S93 in EAAT1 were also changed to alanine and these mutant transporters displayed a similar phenotype to the mutant transporters in Glt_Ph_ in that the ability of Na^+^ to support glutamate transport was substantially reduced.

Why hasn't Na3 been observed in any of the crystal structures of Glt_Ph_? The resolution of the available Glt_Ph_ structures is not good enough to visualise Na^+^ ions directly and the heavy atom thallium (Tl^+^) was used to probe cation binding sites on Glt_Ph_. Two Tl^+^ sites were identified and ion competition studies confirmed that Na1 and Na2 were genuine Na^+^ sites, but Tl^+^ binding was not observed at the Na3 site under these conditions [9]. The Na^+^ affinity for Na3 is considerably higher than for Na1 and Na2 as suggested by the binding free energies (Table 2) and as Tl^+^ has a larger ionic radius than Na^+^ (1.40 vs. 0.95 Å), it simply may not be able to bind to the Na3 site. In addition, the pathway between Na1 and Na3 is narrow [20] and as Tl^+^ was soaked into Glt_Ph_ crystals that already contained Na^+^, exchange between Na^+^ and Tl^+^ may not have been possible at the Na3 site. We have compared the Na3 site of Glt_Ph_ identified in this study (Figure 6A) with the Na^+^ binding site on an unrelated protein, the F-type Na^+^-ATPase from *Ilyobacter tartaricus* [27]. The F-type Na^+^-ATPase is a molecular motor that utilises a Na^+^ gradient to synthesise ATP. The Na^+^ binding sites (11 identical sites) are located on the rotor ring (or c ring) and investigation of these Na^+^ sites (Figure 6C) reveals that they share a number of similarities with Na3 site in Glt_Ph_ (Figure 6A). Both sites are formed by the side chain oxygen of an acidic amino acid (D312 in Glt_Ph_; E65 in c ring), a polar amino acid (N310 in Glt_Ph_; Gln32 in c ring), a backbone carbonyl oxygen (Y89 in Glt_Ph_; Val63 in c ring) and the hydroxyl oxygen of a serine residue (S93 in Glt_Ph_; S66 in c ring). The Na3 binding site in Glt_Ph_ has an additional coordinating ligand from T92. The striking similarities between these two Na^+^ binding sites on proteins unrelated in sequence or function further supports the correct identification of the third Na^+^ binding site of Glt_Ph_ in this study.

Two different orders for Na^+^ and aspartate binding to Glt_Ph_ have been proposed where aspartate binds either before or after Na1 [20]. The binding free energy calculations for Na^+^ and MD simulations of aspartate binding to Glt_Ph_ presented in this study suggest an order of binding where Na3 binds first, followed by Na1. The binding of these two Na^+^ ions creates a favourable site for aspartate to bind. Finally, Na^+^ binding to the Na2 site secures HP2 down over the substrate and results in the ‘occluded’ state observed in previous crystal structures (Figure 6D,E) [8], [9]. The transport domain with aspartate and Na1-3 bound is then predicted to undergo a conformational change and move to the intracellular side of the membrane where it is ready to release its cargo into the cell [26]. Interestingly, examination of the Na3 site in the ‘inward occluded’ structure [26] reveals that two of the coordinating ligands for Na3 (the side chains of N310 and D312) appear to be facing away from this site (Figure 6B). The resolution of this structure is moderate (∼3.8 Å) and side chain placement may not be accurate, but if these movements do occur, the affinity of Na^+^ at the Na3 site in the intracellular occluded state would be reduced and may facilitate unbinding of Na^+^. Further molecular dynamics simulations of the ‘inward occluded’ structure and/or a structure of an inward facing ‘apo’ state of Glt_Ph_ are required to confirm these side chain movements and will shed light on the process of Na^+^ and substrate unbinding.

Three dimensional crystal structures of prokaryotic homologues of membrane proteins have resulted in a major advance in our understanding of the structure and mechanism of their human counterparts. Here, we present the precise location of the third Na^+^ site in Glt_Ph_ and show that this site is conserved in EAAT1. This information is required to develop accurate homology models of the EAATs which will provide useful starting points to investigate the transport mechanism of the human glutamate transporters. Such studies may help to explain the differences between Glt_Ph_ and the EAATs including differences in transport rates, substrate selectivity and K^+^ dependence of transport.

## Materials and Methods

### Ethics statement

Frogs used in this study were anaesthetised to minimize suffering and all surgical procedures followed a protocol approved by The University of Sydney Animal Ethics Committee (protocol # K21/2-2010/3/5269) under the Australian Code of Practice for the Care and Use of Animals for Scientific Purposes.

### Model system and MD simulations

The simulation systems are prepared using the software VMD [28]. The crystal structure of the Glt_Ph_ trimer is embedded in a 1-palmitoyl-2-oleoyl-phosphatidylethanolamine (POPE) phospholipid bilayer and solvated in a box of water molecules with physiological concentration of NaCl. The final system consists of the trimer, 239 lipid molecules, 15688 water molecules and 35 Na^+^ and 35 Cl^−^ ions. Glt_Ph_ has a net charge of +6e, so to keep the system neutral 6 Cl^−^ ions are added in the apo system (more Cl^−^ ions are added when the bound Na^+^ ions are included to preserve neutrality). After the system is built, it is equilibrated in two stages: first, the coordinates of the protein atoms are fixed and the system is equilibrated with 1 atm pressure coupling until the correct water and lipid densities are obtained. The x and y-dimensions of the simulation box are then fixed (at 115 and 113 Å, respectively), and pressure coupling is applied in the z-direction (the average z length is 71 Å). In the second stage, the protein is gradually relaxed in 2.4 ns MD simulations by reducing the restraints on the protein atoms in several steps. The system is further equilibrated for 5 ns with only a small (0.1 kcal/mol/Å^2^) restraint left on the backbone atoms of the protein. This helps to preserve the structural integrity of the protein during long MD simulations. This procedure is repeated for both the closed and open structures, and the final states obtained have been used in all subsequent MD simulations and free energy perturbation (FEP) calculations.

MD simulations are performed using the NAMD package, version 2.7b2 [29] with the CHARMM22 force field [30] including the CMAP corrections [31]. The temperature is kept at 300 K using the Langevin damping method, and the pressure is kept at 1atm using the Langevin piston method. The Lennard-Jones interactions are switched off for distances over 12 Å with a switching distance of 10 Å. Periodic boundary conditions with the particle-mesh Ewald method are employed to calculate the electrostatic interactions without truncation. A time step of 2 fs is used in all MD simulations.

### Free energy calculations

The binding free energies of Na^+^ ions are calculated using the expression, ΔG_b_ = ΔG_int_+ΔG_tr_ , where the first term gives the free energy difference for the interactions of the ion in the binding site and bulk (i.e. translocation energy) and the second term measures the free energy loss due to reduction in translational entropy upon binding [32]. The latter can be estimated from the rms fluctuations of the ion in the binding site (σ_x_, σ_y_,σ_z_) as ΔG_tr_ = −k_B_T ln [(2πe)^3/2^ σ_x_ σ_y_σ_z_/V_0_], where V_0_ = 1660 Å^3^ which is the reference volume for the standard concentration [33]. The interaction energy is calculated using both the free energy perturbation (FEP) and the thermodynamic integration (TI) methods [34], [35]. Because the two methods have yielded essentially the same results, here we report only the TI calculations. Initially, the Na^+^ ion is placed at the appropriate binding position in the protein and the system is equilibrated. In the forward calculation, the bound Na^+^ ion is alchemically transformed to a water molecule while a water molecule in the bulk is transformed to a Na^+^ ion simultaneously. After equilibrating the last window in the forward calculation, a backward calculation is performed, where the opposite transformations are performed bringing the system back to the initial state with a bound Na^+^ ion. Any significant difference between the forward and backward calculations points to hysteresis effects in the TI calculations. Otherwise the binding free energy of the ion is determined from the average of the forward and backward calculations.

Integrals in the TI calculations are evaluated using a seven-point Gaussian quadrature, which has been shown to be sufficiently accurate for calculation of the binding free energies of ions [34]. The simulation systems for the seven windows are adapted from the parallel FEP calculations because the windows are more closely spaced in FEP and hence equilibrate faster. Each window is equilibrated for 0.5 ns followed by a 1-ns production run. Convergence of the free energy results are checked from the running averages which become flat once sufficient sampling is obtained.

The same procedure is used to calculate the binding free energy of the Na^+^ ions at the Na1 and Na3 sites in the T92A and S93A mutations of the transporter. The mutations are implemented using the MUTATOR plug-in from the VMD software, where the side chain of the chosen residue is mutated while the backbone coordinates remain the same. To make sure that the mutated residue is in a stable conformation, the resulting structure is energy minimized and equilibrated for 3 ns in MD simulations before starting the free energy calculations.

### Site-directed Mutagenesis

Site-directed mutagenesis was performed using a polymerase chain reaction (PCR) based method [36] and Velocity DNA polymerase (Bioline) All mutations were sequenced on both strands by Dye Terminator Cycle Sequencing (ABI PRISM, PerkinElmer Life Sciences). The wild type EAAT1 and mutant transporter cDNAs were linearized with SpeI and cRNA transcribed with T7 RNA polymerase using the mMessage mMachine T7 kit (Ambion Inc.).

### Protein purification and reconstitution

Glt_Ph_ protein was purified as described previously [10]. Briefly, membranes were isolated, solubilized with (40 mM) n-dodecyl-β-D-maltopyranoside (C_12_M, Anatrace), and protein was purified using Ni-NTA resin (Qiagen). The C_12_M concentration in the buffer was reduced to 2 mM before addition to Ni-NTA beads. The histidine tag was subsequently removed by digestion with thrombin (10 U/mg protein) and the protein further purified on a size exclusion column where the detergent was exchanged to 7 mM n-decyl-β-D-maltopyranoside (C_10_M, Anatrace).

Pure protein was reconstituted into liposomes using a method modified from Gaillard et al., 1996 [37]. *Escherichia coli* polar lipids and 1-palmitoyl-2-oleoyl-*sn*-glycero-3-phosphocholine (Avanti Polar Lipids), at a ratio of 3∶1, were mixed, dried under nitrogen, and resuspended in internal buffer (100 mM KCl, 20 mM HEPES pH 7.5 or the appropriate internal buffer as indicated below). The lipid mixture was briefly sonicated using a cylindrical sonicator (Labatory Supplies Co.) and the lipid suspension was frozen in liquid nitrogen and thawed at 45°C several times. Liposomes were formed by extrusion through 400 nm polycarbonate membranes (Avanti Polar Lipids) and were treated with Triton X-100 at a 0.5∶1 (w/w) detergent to lipid ratio prior to the addition of protein at 0.25 µg protein/mg lipid [11]. The protein/lipid mixture was left at room temperature for 30 minutes before detergent was removed using SM2 Biobeads (Biorad). The protein/lipid mixture was incubated, with gentle agitation, with three consecutive batches of the Biobeads (80 mg/ml). Proteoliposomes were concentrated by centrifugation at 150,000 g for 30 minutes in a Beckman Optima TLX centrifuge, resuspended at 100 mg lipid/mL and either used immediately or flash frozen in liquid nitrogen and stored at −80°C.

### Proteoliposome transport assay

^3^H-L-aspartate transport by wild type Glt_Ph_ and mutant Glt_Ph_ transporters was assayed using a protocol modified from Gaillard et al., 1996 [37]. Proteoliposomes were loaded with internal buffer (100 mM KCl, 20 mM HEPES pH 7.5) by several freeze/thaw cycles followed by extrusion as described above. The uptake reaction was initiated by diluting the proteoliposomes (100 mg lipid/mL) 133 fold into reaction buffer pre-warmed to 30°C. The reaction buffer contained: 100 mM NaCl, 20 mM HEPES pH 7.5, 1 µM valinomycin and the indicated concentrations of ^3^H-L-aspartate. At each time point, a 200 µL aliquot was removed and diluted 10 fold into ice cold quench buffer (100 mM LiCl, 20 mM HEPES pH 7.5), followed by immediate filtration over nitrocellulose filters (0.22 µm pore size, Millipore). The filters were washed once with 2 mL of ice cold quench buffer and assayed for radioactivity using a Trilux beta counter (Perkin Elmer). The Na^+^ dependence of ^3^H-L-aspartate transport was measured by varying extraliposomal Na^+^ from 0.1 to 300 mM Na^+^. Choline (Ch^+^) was used to balance osmolarity and the intraliposomal K^+^ concentration was adjusted to 300 mM. ^3^H-L-aspartate concentrations used for the Na^+^ titrations were 100 nM for Glt_Ph_ and T92A and 500 nM for S93A; concentrations close to the aspartate *K*_0.5_ value for each transporter. Background levels of uptake were measured by diluting proteoliposomes into internal buffer (100 mM KCl, 20 mM HEPES pH 7.5) containing 1 µM valinomycin and the indicated concentrations of ^3^H-L-aspartate.

### Electrophysiology

All chemicals were obtained from Sigma unless otherwise stated. Stage V oocytes were harvested from *Xenopus laevis* as described previously [38]. 4 ng of cRNA was injected into oocytes and incubated in standard frog Ringer's solution (ND96: 96 mM NaCl, 2 mM KCl, 1 mM MgCl_2_, 1.8 mM CaCl_2_, 5 mM HEPES, pH 7.5) supplemented with 50 µg/mL gentamycin, 2.5 mM sodium pyruvate, 0.5 mM theophylline at 16–18°C.

Current recordings were made using the two-electrode voltage clamp technique with a Geneclamp 500 amplifier (Axon Instruments, Foster City, CA) interfaced with a PowerLab 2/20 chart recorder (ADI Instruments, Sydney, Australia) using the chart software and a Digidata 1322A (Axon Instruments) controlled by an IBM-compatible computer using the pClamp software (version 10, Molecular Devices, Union City, California). When measuring Na^+^ dependence of substrate induced currents, saturating concentrations of L-glutamate were used (300 µM for EAAT1 and 1 mM for T130A and T131A) and the concentration of Na^+^ varied from 1 to 150 mM. NMDG^+^ was used as a Na^+^ substitute to maintain ionic strength of the buffers.

### Analysis of kinetic data

Substrate responses were fitted by least squares as a function of substrate concentration to I/I_max_ = [S]/([S]+*K*_0.5_), where I is the current, I_max_ is the maximal current, *K*_0.5_ is the concentration of substrate that generates half-maximal response, and [S] is the substrate concentration. Na^+^ concentration responses were fit to the Hill equation I/I_max_ = [S]^n^/([S]^n^+(*K*_0.5_)^n^) where n is the Hill coefficient and all other terms are as described above. For ^3^H-L-aspartate uptake by Glt_Ph_, initial rates were calculated from the linear portion of the curve and all data represent the mean ± s.e.m. of at least 3 experiments.

## Supporting Information

Figure S1 **TI calculations for Na^+^.** Convergence of the binding free energy of a Na^+^ ion to the Na3 site is demonstrated using the running averages of the free energies, which flatten out as the data are accumulated. Binding free energies for the negative of the forward (binding site → bulk) and the backward transitions are shown with solid and dashed lines, respectively. The final results read from the end points of the curves, are −22.3 kcal/mol (forward) and −24.2 kcal/mol (backward), whose average gives the interaction energy value (ΔG_int_) quoted in Table 2.
(TIF)

Figure S2 **Maximal velocity of ^3^H-L-aspartate transport.** The maximal rate of transport in the presence of saturating aspartate concentrations and 100 mM NaCl for Glt_Ph_ (black), T92A (white) and S93A (grey).
(TIF)
