# Supplementary figures and images for: Position of the Third Na+ Site in the Aspartate Transporter GltPh and the Human Glutamate Transporter, EAAT1
Source: PLoS One. 2012 Mar 13;7(3):e33058. doi: 10.1371/journal.pone.0033058 (PMC3302783; doi:10.1371/journal.pone.0033058)

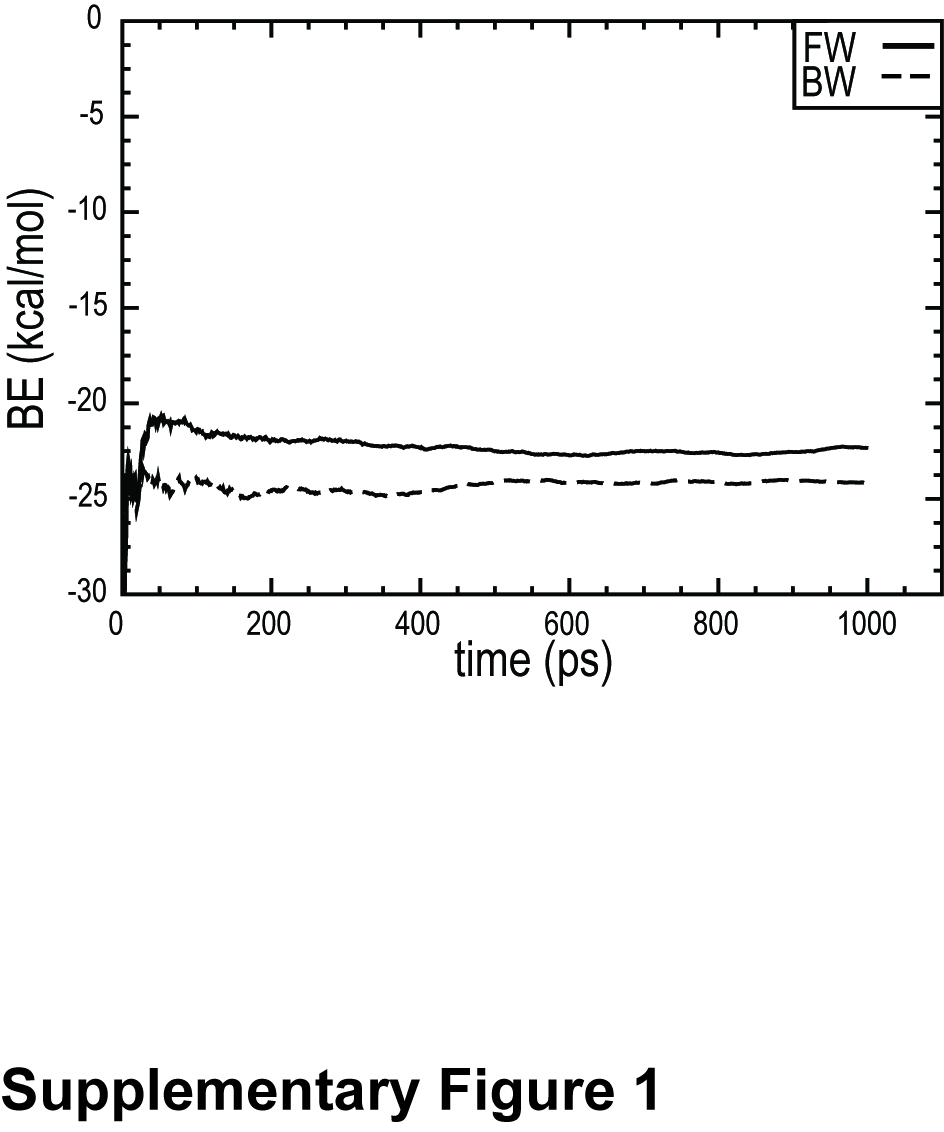

Supplement: Figure S1 — TI calculations for Na+. Convergence of the binding free energy of a Na+ ion to the Na3 site is demonstrated using the running averages of the free energies, which flatten out as the data are accumulated. Binding free energies for the negative of the forward (binding site → bulk) and the backward transitions are shown with solid and dashed lines, respectively. The final results read from the end points of the curves, are −22.3 kcal/mol (forward) and −24.2 kcal/mol (backward), whose average gives the interaction energy value (ΔGint) quoted in Table 2. (TIF) [file pone.0033058.s001.tif]

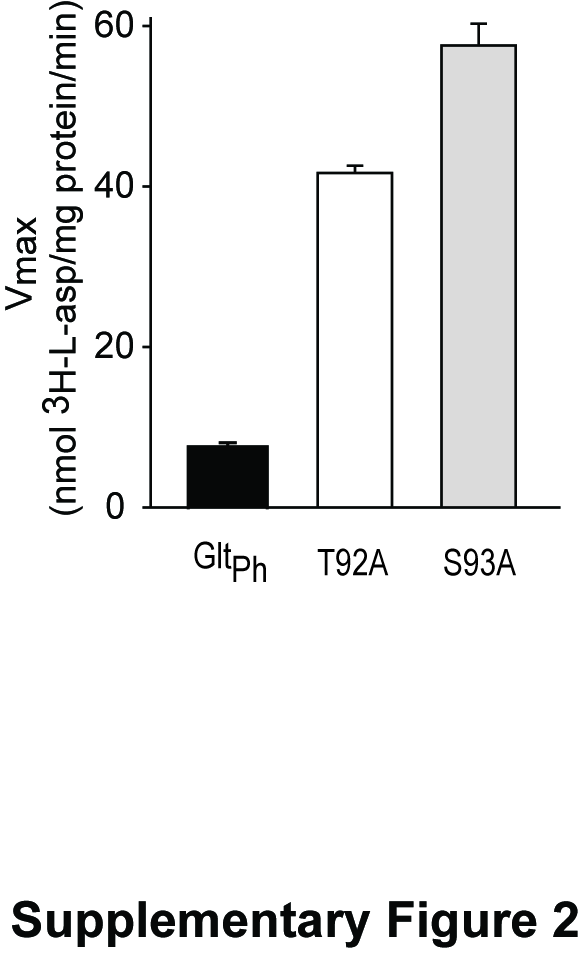

Supplement: Figure S2 — Maximal velocity of 3H-L-aspartate transport. The maximal rate of transport in the presence of saturating aspartate concentrations and 100 mM NaCl for GltPh (black), T92A (white) and S93A (grey). (TIF) [file pone.0033058.s002.tif]
